# Supplementary material for: Optimal minimal residual disease threshold in pediatric acute myeloid leukemia: A retrospective cohort study based on the TARGET database
Source: PLoS Med. 2026 May 8;23(5):e1005088. doi: 10.1371/journal.pmed.1005088 (PMC13155632; doi:10.1371/journal.pmed.1005088)
Supplement: S1 Code — (ZIP) [file pmed.1005088.s002.zip › S2 code/PROJ8_18_tbl1/PROJ8_18_tbl1.htm]

## Kaplan Meier Survival Curve

Outcome: First Event
Time: EFS(years)
Landmark analysis for time segment: EFS(years) <= 5
Log rank test: implements the G-rho family of Harrington and Fleming (1982), with weights on each death of S(t)^rho, where S is the Kaplan-Meier estimate of survival. With rho = 0 this is the log-rank or Mantel-Haenszel test.

|  |  |  |  |
| --- | --- | --- | --- |
|  | N | Observed | Expected |
| factor(X24)=0 | 1073 | 494 | 569.4177 |
| factor(X24)=1 | 132 | 109 | 33.5823 |

Chisq=
180.9988
on
1
degree of freedom, p=
<0.0001
Survival table

|  |  |  |  |  |  |  |  |
| --- | --- | --- | --- | --- | --- | --- | --- |
|  | EFS(years) | N.Risk | N.Event | N.Censor | Survival | 95%CI Low | 95%CI Upp |
| factor(X24)=0 | 0.16 | 1073 | 1 | 0 | 0.9991 | 0.9972 | 1.0000 |
| factor(X24)=0 | 0.17 | 1072 | 2 | 0 | 0.9972 | 0.9940 | 1.0000 |
| factor(X24)=0 | 0.18 | 1070 | 4 | 0 | 0.9935 | 0.9887 | 0.9983 |
| factor(X24)=0 | 0.19 | 1066 | 2 | 1 | 0.9916 | 0.9862 | 0.9971 |
| factor(X24)=0 | 0.2 | 1063 | 5 | 0 | 0.9869 | 0.9802 | 0.9938 |
| factor(X24)=0 | 0.21 | 1058 | 5 | 0 | 0.9823 | 0.9744 | 0.9902 |
| factor(X24)=0 | 0.22 | 1053 | 2 | 0 | 0.9804 | 0.9722 | 0.9887 |
| factor(X24)=0 | 0.23 | 1051 | 2 | 1 | 0.9786 | 0.9699 | 0.9873 |
| factor(X24)=0 | 0.24 | 1048 | 1 | 0 | 0.9776 | 0.9688 | 0.9865 |
| factor(X24)=0 | 0.25 | 1047 | 1 | 0 | 0.9767 | 0.9677 | 0.9858 |
| factor(X24)=0 | 0.26 | 1046 | 4 | 0 | 0.9730 | 0.9633 | 0.9827 |
| factor(X24)=0 | 0.27 | 1042 | 3 | 1 | 0.9701 | 0.9600 | 0.9804 |
| factor(X24)=0 | 0.28 | 1038 | 4 | 0 | 0.9664 | 0.9557 | 0.9773 |
| factor(X24)=0 | 0.29 | 1034 | 3 | 0 | 0.9636 | 0.9525 | 0.9749 |
| factor(X24)=0 | 0.31 | 1031 | 1 | 0 | 0.9627 | 0.9514 | 0.9741 |
| factor(X24)=0 | 0.32 | 1030 | 3 | 0 | 0.9599 | 0.9482 | 0.9717 |
| factor(X24)=0 | 0.33 | 1027 | 3 | 1 | 0.9571 | 0.9450 | 0.9693 |
| factor(X24)=0 | 0.35 | 1023 | 2 | 0 | 0.9552 | 0.9429 | 0.9677 |
| factor(X24)=0 | 0.36 | 1021 | 1 | 0 | 0.9543 | 0.9418 | 0.9669 |
| factor(X24)=0 | 0.37 | 1020 | 3 | 0 | 0.9515 | 0.9387 | 0.9644 |
| factor(X24)=0 | 0.38 | 1017 | 2 | 0 | 0.9496 | 0.9366 | 0.9628 |
| factor(X24)=0 | 0.4 | 1015 | 4 | 0 | 0.9458 | 0.9324 | 0.9595 |
| factor(X24)=0 | 0.41 | 1011 | 4 | 0 | 0.9421 | 0.9282 | 0.9562 |
| factor(X24)=0 | 0.42 | 1007 | 3 | 0 | 0.9393 | 0.9251 | 0.9537 |
| factor(X24)=0 | 0.43 | 1004 | 1 | 0 | 0.9384 | 0.9241 | 0.9529 |
| factor(X24)=0 | 0.44 | 1003 | 5 | 0 | 0.9337 | 0.9189 | 0.9487 |
| factor(X24)=0 | 0.45 | 998 | 3 | 0 | 0.9309 | 0.9158 | 0.9462 |
| factor(X24)=0 | 0.46 | 995 | 2 | 0 | 0.9290 | 0.9137 | 0.9445 |
| factor(X24)=0 | 0.47 | 993 | 3 | 0 | 0.9262 | 0.9107 | 0.9420 |
| factor(X24)=0 | 0.48 | 990 | 2 | 0 | 0.9243 | 0.9086 | 0.9403 |
| factor(X24)=0 | 0.5 | 988 | 3 | 0 | 0.9215 | 0.9055 | 0.9378 |
| factor(X24)=0 | 0.51 | 985 | 1 | 0 | 0.9206 | 0.9045 | 0.9369 |
| factor(X24)=0 | 0.52 | 984 | 5 | 0 | 0.9159 | 0.8994 | 0.9327 |
| factor(X24)=0 | 0.53 | 979 | 7 | 0 | 0.9094 | 0.8923 | 0.9267 |
| factor(X24)=0 | 0.54 | 972 | 3 | 0 | 0.9065 | 0.8893 | 0.9242 |
| factor(X24)=0 | 0.55 | 969 | 5 | 0 | 0.9019 | 0.8842 | 0.9199 |
| factor(X24)=0 | 0.56 | 964 | 7 | 0 | 0.8953 | 0.8772 | 0.9139 |
| factor(X24)=0 | 0.57 | 957 | 5 | 0 | 0.8906 | 0.8721 | 0.9095 |
| factor(X24)=0 | 0.58 | 952 | 5 | 0 | 0.8860 | 0.8671 | 0.9052 |
| factor(X24)=0 | 0.59 | 947 | 4 | 0 | 0.8822 | 0.8631 | 0.9018 |
| factor(X24)=0 | 0.6 | 943 | 5 | 0 | 0.8775 | 0.8581 | 0.8974 |
| factor(X24)=0 | 0.61 | 938 | 1 | 0 | 0.8766 | 0.8571 | 0.8965 |
| factor(X24)=0 | 0.62 | 937 | 2 | 0 | 0.8747 | 0.8551 | 0.8948 |
| factor(X24)=0 | 0.63 | 935 | 1 | 0 | 0.8738 | 0.8541 | 0.8939 |
| factor(X24)=0 | 0.64 | 934 | 2 | 0 | 0.8719 | 0.8521 | 0.8922 |
| factor(X24)=0 | 0.65 | 932 | 4 | 0 | 0.8682 | 0.8481 | 0.8887 |
| factor(X24)=0 | 0.66 | 928 | 2 | 0 | 0.8663 | 0.8462 | 0.8870 |
| factor(X24)=0 | 0.67 | 926 | 3 | 0 | 0.8635 | 0.8432 | 0.8843 |
| factor(X24)=0 | 0.68 | 923 | 8 | 0 | 0.8560 | 0.8352 | 0.8773 |
| factor(X24)=0 | 0.69 | 915 | 5 | 0 | 0.8513 | 0.8303 | 0.8729 |
| factor(X24)=0 | 0.7 | 910 | 7 | 0 | 0.8448 | 0.8234 | 0.8668 |
| factor(X24)=0 | 0.71 | 903 | 4 | 0 | 0.8411 | 0.8194 | 0.8633 |
| factor(X24)=0 | 0.72 | 899 | 7 | 0 | 0.8345 | 0.8125 | 0.8571 |
| factor(X24)=0 | 0.73 | 892 | 4 | 0 | 0.8308 | 0.8086 | 0.8535 |
| factor(X24)=0 | 0.74 | 888 | 4 | 0 | 0.8270 | 0.8047 | 0.8500 |
| factor(X24)=0 | 0.75 | 884 | 4 | 0 | 0.8233 | 0.8007 | 0.8465 |
| factor(X24)=0 | 0.76 | 880 | 10 | 0 | 0.8139 | 0.7909 | 0.8376 |
| factor(X24)=0 | 0.77 | 870 | 1 | 0 | 0.8130 | 0.7900 | 0.8367 |
| factor(X24)=0 | 0.78 | 869 | 4 | 1 | 0.8092 | 0.7860 | 0.8331 |
| factor(X24)=0 | 0.79 | 864 | 8 | 0 | 0.8018 | 0.7782 | 0.8260 |
| factor(X24)=0 | 0.8 | 856 | 4 | 0 | 0.7980 | 0.7743 | 0.8224 |
| factor(X24)=0 | 0.81 | 852 | 8 | 0 | 0.7905 | 0.7665 | 0.8153 |
| factor(X24)=0 | 0.82 | 844 | 9 | 0 | 0.7821 | 0.7577 | 0.8072 |
| factor(X24)=0 | 0.83 | 835 | 4 | 0 | 0.7783 | 0.7538 | 0.8036 |
| factor(X24)=0 | 0.84 | 831 | 9 | 0 | 0.7699 | 0.7451 | 0.7956 |
| factor(X24)=0 | 0.85 | 822 | 2 | 0 | 0.7680 | 0.7431 | 0.7938 |
| factor(X24)=0 | 0.86 | 820 | 3 | 0 | 0.7652 | 0.7402 | 0.7911 |
| factor(X24)=0 | 0.87 | 817 | 2 | 0 | 0.7634 | 0.7383 | 0.7893 |
| factor(X24)=0 | 0.88 | 815 | 8 | 0 | 0.7559 | 0.7305 | 0.7821 |
| factor(X24)=0 | 0.89 | 807 | 2 | 0 | 0.7540 | 0.7286 | 0.7803 |
| factor(X24)=0 | 0.9 | 805 | 4 | 0 | 0.7502 | 0.7247 | 0.7766 |
| factor(X24)=0 | 0.91 | 801 | 5 | 0 | 0.7456 | 0.7199 | 0.7721 |
| factor(X24)=0 | 0.92 | 796 | 5 | 0 | 0.7409 | 0.7151 | 0.7676 |
| factor(X24)=0 | 0.93 | 791 | 9 | 0 | 0.7324 | 0.7064 | 0.7595 |
| factor(X24)=0 | 0.94 | 782 | 2 | 0 | 0.7306 | 0.7045 | 0.7577 |
| factor(X24)=0 | 0.95 | 780 | 1 | 0 | 0.7296 | 0.7035 | 0.7568 |
| factor(X24)=0 | 0.96 | 779 | 2 | 1 | 0.7278 | 0.7016 | 0.7549 |
| factor(X24)=0 | 0.97 | 776 | 3 | 0 | 0.7249 | 0.6987 | 0.7522 |
| factor(X24)=0 | 0.98 | 773 | 1 | 0 | 0.7240 | 0.6977 | 0.7513 |
| factor(X24)=0 | 0.99 | 772 | 4 | 1 | 0.7203 | 0.6938 | 0.7477 |
| factor(X24)=0 | 1 | 767 | 4 | 0 | 0.7165 | 0.6900 | 0.7440 |
| factor(X24)=0 | 1.01 | 763 | 3 | 0 | 0.7137 | 0.6871 | 0.7413 |
| factor(X24)=0 | 1.02 | 760 | 3 | 1 | 0.7109 | 0.6842 | 0.7386 |
| factor(X24)=0 | 1.03 | 756 | 3 | 0 | 0.7080 | 0.6813 | 0.7358 |
| factor(X24)=0 | 1.05 | 753 | 6 | 0 | 0.7024 | 0.6755 | 0.7304 |
| factor(X24)=0 | 1.06 | 747 | 3 | 1 | 0.6996 | 0.6726 | 0.7276 |
| factor(X24)=0 | 1.07 | 743 | 4 | 0 | 0.6958 | 0.6688 | 0.7240 |
| factor(X24)=0 | 1.08 | 739 | 6 | 0 | 0.6902 | 0.6630 | 0.7185 |
| factor(X24)=0 | 1.09 | 733 | 2 | 0 | 0.6883 | 0.6611 | 0.7166 |
| factor(X24)=0 | 1.1 | 731 | 2 | 0 | 0.6864 | 0.6591 | 0.7148 |
| factor(X24)=0 | 1.11 | 729 | 1 | 2 | 0.6855 | 0.6582 | 0.7139 |
| factor(X24)=0 | 1.12 | 726 | 4 | 1 | 0.6817 | 0.6543 | 0.7102 |
| factor(X24)=0 | 1.13 | 721 | 4 | 0 | 0.6779 | 0.6504 | 0.7065 |
| factor(X24)=0 | 1.14 | 717 | 2 | 1 | 0.6760 | 0.6485 | 0.7047 |
| factor(X24)=0 | 1.15 | 714 | 1 | 1 | 0.6751 | 0.6475 | 0.7038 |
| factor(X24)=0 | 1.16 | 712 | 2 | 0 | 0.6732 | 0.6456 | 0.7019 |
| factor(X24)=0 | 1.17 | 710 | 2 | 0 | 0.6713 | 0.6437 | 0.7001 |
| factor(X24)=0 | 1.18 | 708 | 2 | 0 | 0.6694 | 0.6417 | 0.6982 |
| factor(X24)=0 | 1.19 | 706 | 2 | 0 | 0.6675 | 0.6398 | 0.6964 |
| factor(X24)=0 | 1.2 | 704 | 5 | 0 | 0.6627 | 0.6350 | 0.6917 |
| factor(X24)=0 | 1.21 | 699 | 1 | 1 | 0.6618 | 0.6340 | 0.6908 |
| factor(X24)=0 | 1.24 | 697 | 1 | 0 | 0.6608 | 0.6330 | 0.6899 |
| factor(X24)=0 | 1.25 | 696 | 2 | 1 | 0.6589 | 0.6311 | 0.6880 |
| factor(X24)=0 | 1.26 | 693 | 1 | 0 | 0.6580 | 0.6301 | 0.6871 |
| factor(X24)=0 | 1.27 | 692 | 4 | 0 | 0.6542 | 0.6263 | 0.6834 |
| factor(X24)=0 | 1.28 | 688 | 2 | 1 | 0.6523 | 0.6243 | 0.6815 |
| factor(X24)=0 | 1.3 | 685 | 4 | 0 | 0.6485 | 0.6204 | 0.6778 |
| factor(X24)=0 | 1.31 | 681 | 2 | 0 | 0.6466 | 0.6185 | 0.6759 |
| factor(X24)=0 | 1.32 | 679 | 1 | 0 | 0.6456 | 0.6175 | 0.6750 |
| factor(X24)=0 | 1.34 | 678 | 3 | 1 | 0.6428 | 0.6146 | 0.6722 |
| factor(X24)=0 | 1.35 | 674 | 3 | 0 | 0.6399 | 0.6117 | 0.6694 |
| factor(X24)=0 | 1.36 | 671 | 3 | 0 | 0.6370 | 0.6088 | 0.6666 |
| factor(X24)=0 | 1.37 | 668 | 2 | 1 | 0.6351 | 0.6069 | 0.6647 |
| factor(X24)=0 | 1.38 | 665 | 3 | 0 | 0.6323 | 0.6040 | 0.6619 |
| factor(X24)=0 | 1.39 | 662 | 4 | 1 | 0.6284 | 0.6001 | 0.6582 |
| factor(X24)=0 | 1.4 | 657 | 1 | 0 | 0.6275 | 0.5991 | 0.6572 |
| factor(X24)=0 | 1.41 | 656 | 1 | 0 | 0.6265 | 0.5981 | 0.6563 |
| factor(X24)=0 | 1.42 | 655 | 2 | 0 | 0.6246 | 0.5962 | 0.6544 |
| factor(X24)=0 | 1.43 | 653 | 4 | 0 | 0.6208 | 0.5923 | 0.6507 |
| factor(X24)=0 | 1.45 | 649 | 2 | 0 | 0.6189 | 0.5904 | 0.6488 |
| factor(X24)=0 | 1.46 | 647 | 1 | 0 | 0.6179 | 0.5894 | 0.6478 |
| factor(X24)=0 | 1.48 | 646 | 2 | 0 | 0.6160 | 0.5875 | 0.6460 |
| factor(X24)=0 | 1.49 | 644 | 3 | 0 | 0.6131 | 0.5846 | 0.6431 |
| factor(X24)=0 | 1.5 | 641 | 2 | 0 | 0.6112 | 0.5826 | 0.6413 |
| factor(X24)=0 | 1.51 | 639 | 1 | 0 | 0.6103 | 0.5816 | 0.6403 |
| factor(X24)=0 | 1.53 | 638 | 1 | 0 | 0.6093 | 0.5807 | 0.6394 |
| factor(X24)=0 | 1.54 | 637 | 1 | 0 | 0.6084 | 0.5797 | 0.6384 |
| factor(X24)=0 | 1.55 | 636 | 3 | 0 | 0.6055 | 0.5768 | 0.6356 |
| factor(X24)=0 | 1.56 | 633 | 1 | 0 | 0.6045 | 0.5758 | 0.6347 |
| factor(X24)=0 | 1.58 | 632 | 5 | 0 | 0.5998 | 0.5710 | 0.6300 |
| factor(X24)=0 | 1.6 | 627 | 2 | 0 | 0.5978 | 0.5691 | 0.6281 |
| factor(X24)=0 | 1.61 | 625 | 0 | 1 | 0.5978 | 0.5691 | 0.6281 |
| factor(X24)=0 | 1.62 | 624 | 2 | 0 | 0.5959 | 0.5671 | 0.6262 |
| factor(X24)=0 | 1.64 | 622 | 1 | 0 | 0.5950 | 0.5662 | 0.6252 |
| factor(X24)=0 | 1.65 | 621 | 2 | 0 | 0.5930 | 0.5642 | 0.6233 |
| factor(X24)=0 | 1.66 | 619 | 1 | 0 | 0.5921 | 0.5633 | 0.6224 |
| factor(X24)=0 | 1.67 | 618 | 1 | 0 | 0.5911 | 0.5623 | 0.6215 |
| factor(X24)=0 | 1.71 | 617 | 0 | 1 | 0.5911 | 0.5623 | 0.6215 |
| factor(X24)=0 | 1.72 | 616 | 3 | 0 | 0.5883 | 0.5594 | 0.6186 |
| factor(X24)=0 | 1.75 | 613 | 0 | 1 | 0.5883 | 0.5594 | 0.6186 |
| factor(X24)=0 | 1.77 | 612 | 1 | 0 | 0.5873 | 0.5584 | 0.6177 |
| factor(X24)=0 | 1.78 | 611 | 1 | 0 | 0.5863 | 0.5574 | 0.6167 |
| factor(X24)=0 | 1.79 | 610 | 1 | 0 | 0.5854 | 0.5565 | 0.6158 |
| factor(X24)=0 | 1.81 | 609 | 1 | 0 | 0.5844 | 0.5555 | 0.6148 |
| factor(X24)=0 | 1.83 | 608 | 0 | 1 | 0.5844 | 0.5555 | 0.6148 |
| factor(X24)=0 | 1.87 | 607 | 1 | 1 | 0.5834 | 0.5545 | 0.6139 |
| factor(X24)=0 | 1.89 | 605 | 2 | 0 | 0.5815 | 0.5526 | 0.6120 |
| factor(X24)=0 | 1.9 | 603 | 2 | 0 | 0.5796 | 0.5506 | 0.6101 |
| factor(X24)=0 | 1.92 | 601 | 2 | 1 | 0.5777 | 0.5487 | 0.6082 |
| factor(X24)=0 | 1.94 | 598 | 1 | 2 | 0.5767 | 0.5477 | 0.6072 |
| factor(X24)=0 | 1.96 | 595 | 0 | 1 | 0.5767 | 0.5477 | 0.6072 |
| factor(X24)=0 | 1.98 | 594 | 4 | 1 | 0.5728 | 0.5438 | 0.6034 |
| factor(X24)=0 | 1.99 | 589 | 1 | 0 | 0.5718 | 0.5428 | 0.6024 |
| factor(X24)=0 | 2.03 | 588 | 1 | 0 | 0.5709 | 0.5418 | 0.6015 |
| factor(X24)=0 | 2.05 | 587 | 1 | 0 | 0.5699 | 0.5408 | 0.6005 |
| factor(X24)=0 | 2.07 | 586 | 1 | 0 | 0.5689 | 0.5399 | 0.5995 |
| factor(X24)=0 | 2.08 | 585 | 1 | 0 | 0.5679 | 0.5389 | 0.5986 |
| factor(X24)=0 | 2.09 | 584 | 2 | 0 | 0.5660 | 0.5369 | 0.5967 |
| factor(X24)=0 | 2.1 | 582 | 0 | 1 | 0.5660 | 0.5369 | 0.5967 |
| factor(X24)=0 | 2.11 | 581 | 1 | 0 | 0.5650 | 0.5359 | 0.5957 |
| factor(X24)=0 | 2.13 | 580 | 1 | 0 | 0.5641 | 0.5350 | 0.5947 |
| factor(X24)=0 | 2.14 | 579 | 1 | 0 | 0.5631 | 0.5340 | 0.5938 |
| factor(X24)=0 | 2.15 | 578 | 1 | 0 | 0.5621 | 0.5330 | 0.5928 |
| factor(X24)=0 | 2.18 | 577 | 0 | 1 | 0.5621 | 0.5330 | 0.5928 |
| factor(X24)=0 | 2.2 | 576 | 1 | 0 | 0.5611 | 0.5320 | 0.5918 |
| factor(X24)=0 | 2.22 | 575 | 2 | 1 | 0.5592 | 0.5300 | 0.5899 |
| factor(X24)=0 | 2.23 | 572 | 1 | 1 | 0.5582 | 0.5291 | 0.5889 |
| factor(X24)=0 | 2.27 | 570 | 0 | 2 | 0.5582 | 0.5291 | 0.5889 |
| factor(X24)=0 | 2.3 | 568 | 1 | 0 | 0.5572 | 0.5281 | 0.5880 |
| factor(X24)=0 | 2.31 | 567 | 1 | 0 | 0.5562 | 0.5271 | 0.5870 |
| factor(X24)=0 | 2.32 | 566 | 0 | 2 | 0.5562 | 0.5271 | 0.5870 |
| factor(X24)=0 | 2.34 | 564 | 1 | 0 | 0.5552 | 0.5261 | 0.5860 |
| factor(X24)=0 | 2.36 | 563 | 1 | 0 | 0.5543 | 0.5251 | 0.5851 |
| factor(X24)=0 | 2.37 | 562 | 1 | 1 | 0.5533 | 0.5241 | 0.5841 |
| factor(X24)=0 | 2.39 | 560 | 0 | 1 | 0.5533 | 0.5241 | 0.5841 |
| factor(X24)=0 | 2.4 | 559 | 0 | 1 | 0.5533 | 0.5241 | 0.5841 |
| factor(X24)=0 | 2.41 | 558 | 0 | 1 | 0.5533 | 0.5241 | 0.5841 |
| factor(X24)=0 | 2.42 | 557 | 1 | 0 | 0.5523 | 0.5231 | 0.5831 |
| factor(X24)=0 | 2.43 | 556 | 0 | 1 | 0.5523 | 0.5231 | 0.5831 |
| factor(X24)=0 | 2.44 | 555 | 0 | 2 | 0.5523 | 0.5231 | 0.5831 |
| factor(X24)=0 | 2.46 | 553 | 0 | 1 | 0.5523 | 0.5231 | 0.5831 |
| factor(X24)=0 | 2.47 | 552 | 0 | 1 | 0.5523 | 0.5231 | 0.5831 |
| factor(X24)=0 | 2.48 | 551 | 1 | 0 | 0.5513 | 0.5221 | 0.5821 |
| factor(X24)=0 | 2.49 | 550 | 0 | 1 | 0.5513 | 0.5221 | 0.5821 |
| factor(X24)=0 | 2.5 | 549 | 0 | 4 | 0.5513 | 0.5221 | 0.5821 |
| factor(X24)=0 | 2.51 | 545 | 1 | 1 | 0.5503 | 0.5211 | 0.5811 |
| factor(X24)=0 | 2.52 | 543 | 1 | 0 | 0.5493 | 0.5200 | 0.5801 |
| factor(X24)=0 | 2.53 | 542 | 1 | 1 | 0.5482 | 0.5190 | 0.5791 |
| factor(X24)=0 | 2.55 | 540 | 0 | 1 | 0.5482 | 0.5190 | 0.5791 |
| factor(X24)=0 | 2.56 | 539 | 0 | 1 | 0.5482 | 0.5190 | 0.5791 |
| factor(X24)=0 | 2.6 | 538 | 0 | 1 | 0.5482 | 0.5190 | 0.5791 |
| factor(X24)=0 | 2.61 | 537 | 1 | 1 | 0.5472 | 0.5180 | 0.5781 |
| factor(X24)=0 | 2.62 | 535 | 2 | 0 | 0.5452 | 0.5159 | 0.5761 |
| factor(X24)=0 | 2.63 | 533 | 0 | 1 | 0.5452 | 0.5159 | 0.5761 |
| factor(X24)=0 | 2.64 | 532 | 0 | 1 | 0.5452 | 0.5159 | 0.5761 |
| factor(X24)=0 | 2.68 | 531 | 0 | 1 | 0.5452 | 0.5159 | 0.5761 |
| factor(X24)=0 | 2.7 | 530 | 1 | 1 | 0.5441 | 0.5149 | 0.5751 |
| factor(X24)=0 | 2.71 | 528 | 0 | 1 | 0.5441 | 0.5149 | 0.5751 |
| factor(X24)=0 | 2.73 | 527 | 0 | 1 | 0.5441 | 0.5149 | 0.5751 |
| factor(X24)=0 | 2.75 | 526 | 0 | 1 | 0.5441 | 0.5149 | 0.5751 |
| factor(X24)=0 | 2.78 | 525 | 0 | 2 | 0.5441 | 0.5149 | 0.5751 |
| factor(X24)=0 | 2.79 | 523 | 0 | 2 | 0.5441 | 0.5149 | 0.5751 |
| factor(X24)=0 | 2.83 | 521 | 1 | 1 | 0.5431 | 0.5138 | 0.5740 |
| factor(X24)=0 | 2.89 | 519 | 0 | 1 | 0.5431 | 0.5138 | 0.5740 |
| factor(X24)=0 | 2.93 | 518 | 0 | 1 | 0.5431 | 0.5138 | 0.5740 |
| factor(X24)=0 | 2.95 | 517 | 0 | 1 | 0.5431 | 0.5138 | 0.5740 |
| factor(X24)=0 | 2.98 | 516 | 0 | 1 | 0.5431 | 0.5138 | 0.5740 |
| factor(X24)=0 | 2.99 | 515 | 1 | 0 | 0.5420 | 0.5128 | 0.5730 |
| factor(X24)=0 | 3 | 514 | 0 | 1 | 0.5420 | 0.5128 | 0.5730 |
| factor(X24)=0 | 3.01 | 513 | 0 | 1 | 0.5420 | 0.5128 | 0.5730 |
| factor(X24)=0 | 3.02 | 512 | 0 | 1 | 0.5420 | 0.5128 | 0.5730 |
| factor(X24)=0 | 3.03 | 511 | 0 | 1 | 0.5420 | 0.5128 | 0.5730 |
| factor(X24)=0 | 3.04 | 510 | 0 | 2 | 0.5420 | 0.5128 | 0.5730 |
| factor(X24)=0 | 3.05 | 508 | 0 | 2 | 0.5420 | 0.5128 | 0.5730 |
| factor(X24)=0 | 3.07 | 506 | 0 | 1 | 0.5420 | 0.5128 | 0.5730 |
| factor(X24)=0 | 3.11 | 505 | 0 | 1 | 0.5420 | 0.5128 | 0.5730 |
| factor(X24)=0 | 3.14 | 504 | 0 | 1 | 0.5420 | 0.5128 | 0.5730 |
| factor(X24)=0 | 3.15 | 503 | 0 | 1 | 0.5420 | 0.5128 | 0.5730 |
| factor(X24)=0 | 3.16 | 502 | 0 | 2 | 0.5420 | 0.5128 | 0.5730 |
| factor(X24)=0 | 3.17 | 500 | 0 | 1 | 0.5420 | 0.5128 | 0.5730 |
| factor(X24)=0 | 3.18 | 499 | 1 | 3 | 0.5410 | 0.5117 | 0.5719 |
| factor(X24)=0 | 3.19 | 495 | 0 | 1 | 0.5410 | 0.5117 | 0.5719 |
| factor(X24)=0 | 3.2 | 494 | 0 | 1 | 0.5410 | 0.5117 | 0.5719 |
| factor(X24)=0 | 3.22 | 493 | 0 | 1 | 0.5410 | 0.5117 | 0.5719 |
| factor(X24)=0 | 3.23 | 492 | 0 | 1 | 0.5410 | 0.5117 | 0.5719 |
| factor(X24)=0 | 3.24 | 491 | 0 | 1 | 0.5410 | 0.5117 | 0.5719 |
| factor(X24)=0 | 3.25 | 490 | 0 | 2 | 0.5410 | 0.5117 | 0.5719 |
| factor(X24)=0 | 3.28 | 488 | 0 | 3 | 0.5410 | 0.5117 | 0.5719 |
| factor(X24)=0 | 3.29 | 485 | 0 | 1 | 0.5410 | 0.5117 | 0.5719 |
| factor(X24)=0 | 3.3 | 484 | 0 | 2 | 0.5410 | 0.5117 | 0.5719 |
| factor(X24)=0 | 3.32 | 482 | 0 | 1 | 0.5410 | 0.5117 | 0.5719 |
| factor(X24)=0 | 3.33 | 481 | 0 | 3 | 0.5410 | 0.5117 | 0.5719 |
| factor(X24)=0 | 3.34 | 478 | 0 | 2 | 0.5410 | 0.5117 | 0.5719 |
| factor(X24)=0 | 3.35 | 476 | 0 | 3 | 0.5410 | 0.5117 | 0.5719 |
| factor(X24)=0 | 3.36 | 473 | 0 | 2 | 0.5410 | 0.5117 | 0.5719 |
| factor(X24)=0 | 3.38 | 471 | 0 | 3 | 0.5410 | 0.5117 | 0.5719 |
| factor(X24)=0 | 3.39 | 468 | 1 | 2 | 0.5398 | 0.5105 | 0.5708 |
| factor(X24)=0 | 3.4 | 465 | 0 | 1 | 0.5398 | 0.5105 | 0.5708 |
| factor(X24)=0 | 3.41 | 464 | 0 | 4 | 0.5398 | 0.5105 | 0.5708 |
| factor(X24)=0 | 3.43 | 460 | 0 | 1 | 0.5398 | 0.5105 | 0.5708 |
| factor(X24)=0 | 3.44 | 459 | 0 | 4 | 0.5398 | 0.5105 | 0.5708 |
| factor(X24)=0 | 3.45 | 455 | 0 | 2 | 0.5398 | 0.5105 | 0.5708 |
| factor(X24)=0 | 3.46 | 453 | 0 | 1 | 0.5398 | 0.5105 | 0.5708 |
| factor(X24)=0 | 3.48 | 452 | 0 | 2 | 0.5398 | 0.5105 | 0.5708 |
| factor(X24)=0 | 3.5 | 450 | 0 | 3 | 0.5398 | 0.5105 | 0.5708 |
| factor(X24)=0 | 3.52 | 447 | 0 | 1 | 0.5398 | 0.5105 | 0.5708 |
| factor(X24)=0 | 3.53 | 446 | 0 | 1 | 0.5398 | 0.5105 | 0.5708 |
| factor(X24)=0 | 3.54 | 445 | 1 | 1 | 0.5386 | 0.5093 | 0.5696 |
| factor(X24)=0 | 3.56 | 443 | 0 | 4 | 0.5386 | 0.5093 | 0.5696 |
| factor(X24)=0 | 3.58 | 439 | 0 | 1 | 0.5386 | 0.5093 | 0.5696 |
| factor(X24)=0 | 3.62 | 438 | 0 | 1 | 0.5386 | 0.5093 | 0.5696 |
| factor(X24)=0 | 3.63 | 437 | 0 | 1 | 0.5386 | 0.5093 | 0.5696 |
| factor(X24)=0 | 3.65 | 436 | 0 | 2 | 0.5386 | 0.5093 | 0.5696 |
| factor(X24)=0 | 3.66 | 434 | 0 | 1 | 0.5386 | 0.5093 | 0.5696 |
| factor(X24)=0 | 3.74 | 433 | 0 | 1 | 0.5386 | 0.5093 | 0.5696 |
| factor(X24)=0 | 3.79 | 432 | 0 | 1 | 0.5386 | 0.5093 | 0.5696 |
| factor(X24)=0 | 3.81 | 431 | 0 | 2 | 0.5386 | 0.5093 | 0.5696 |
| factor(X24)=0 | 3.83 | 429 | 0 | 1 | 0.5386 | 0.5093 | 0.5696 |
| factor(X24)=0 | 3.84 | 428 | 0 | 2 | 0.5386 | 0.5093 | 0.5696 |
| factor(X24)=0 | 3.87 | 426 | 0 | 1 | 0.5386 | 0.5093 | 0.5696 |
| factor(X24)=0 | 3.88 | 425 | 0 | 1 | 0.5386 | 0.5093 | 0.5696 |
| factor(X24)=0 | 3.89 | 424 | 0 | 1 | 0.5386 | 0.5093 | 0.5696 |
| factor(X24)=0 | 3.9 | 423 | 0 | 2 | 0.5386 | 0.5093 | 0.5696 |
| factor(X24)=0 | 3.95 | 421 | 0 | 1 | 0.5386 | 0.5093 | 0.5696 |
| factor(X24)=0 | 3.96 | 420 | 0 | 1 | 0.5386 | 0.5093 | 0.5696 |
| factor(X24)=0 | 3.97 | 419 | 0 | 1 | 0.5386 | 0.5093 | 0.5696 |
| factor(X24)=0 | 3.99 | 418 | 1 | 0 | 0.5373 | 0.5080 | 0.5684 |
| factor(X24)=0 | 4.04 | 417 | 0 | 2 | 0.5373 | 0.5080 | 0.5684 |
| factor(X24)=0 | 4.05 | 415 | 0 | 2 | 0.5373 | 0.5080 | 0.5684 |
| factor(X24)=0 | 4.06 | 413 | 0 | 2 | 0.5373 | 0.5080 | 0.5684 |
| factor(X24)=0 | 4.07 | 411 | 1 | 1 | 0.5360 | 0.5066 | 0.5671 |
| factor(X24)=0 | 4.08 | 409 | 0 | 2 | 0.5360 | 0.5066 | 0.5671 |
| factor(X24)=0 | 4.13 | 407 | 0 | 2 | 0.5360 | 0.5066 | 0.5671 |
| factor(X24)=0 | 4.15 | 405 | 0 | 2 | 0.5360 | 0.5066 | 0.5671 |
| factor(X24)=0 | 4.16 | 403 | 0 | 1 | 0.5360 | 0.5066 | 0.5671 |
| factor(X24)=0 | 4.19 | 402 | 0 | 2 | 0.5360 | 0.5066 | 0.5671 |
| factor(X24)=0 | 4.22 | 400 | 0 | 2 | 0.5360 | 0.5066 | 0.5671 |
| factor(X24)=0 | 4.23 | 398 | 1 | 0 | 0.5347 | 0.5052 | 0.5658 |
| factor(X24)=0 | 4.26 | 397 | 0 | 1 | 0.5347 | 0.5052 | 0.5658 |
| factor(X24)=0 | 4.27 | 396 | 0 | 2 | 0.5347 | 0.5052 | 0.5658 |
| factor(X24)=0 | 4.31 | 394 | 0 | 2 | 0.5347 | 0.5052 | 0.5658 |
| factor(X24)=0 | 4.32 | 392 | 0 | 1 | 0.5347 | 0.5052 | 0.5658 |
| factor(X24)=0 | 4.33 | 391 | 0 | 1 | 0.5347 | 0.5052 | 0.5658 |
| factor(X24)=0 | 4.34 | 390 | 0 | 1 | 0.5347 | 0.5052 | 0.5658 |
| factor(X24)=0 | 4.35 | 389 | 0 | 4 | 0.5347 | 0.5052 | 0.5658 |
| factor(X24)=0 | 4.36 | 385 | 0 | 4 | 0.5347 | 0.5052 | 0.5658 |
| factor(X24)=0 | 4.38 | 381 | 0 | 3 | 0.5347 | 0.5052 | 0.5658 |
| factor(X24)=0 | 4.4 | 378 | 0 | 1 | 0.5347 | 0.5052 | 0.5658 |
| factor(X24)=0 | 4.41 | 377 | 0 | 2 | 0.5347 | 0.5052 | 0.5658 |
| factor(X24)=0 | 4.42 | 375 | 0 | 2 | 0.5347 | 0.5052 | 0.5658 |
| factor(X24)=0 | 4.44 | 373 | 0 | 3 | 0.5347 | 0.5052 | 0.5658 |
| factor(X24)=0 | 4.45 | 370 | 0 | 1 | 0.5347 | 0.5052 | 0.5658 |
| factor(X24)=0 | 4.47 | 369 | 0 | 2 | 0.5347 | 0.5052 | 0.5658 |
| factor(X24)=0 | 4.48 | 367 | 0 | 2 | 0.5347 | 0.5052 | 0.5658 |
| factor(X24)=0 | 4.49 | 365 | 0 | 2 | 0.5347 | 0.5052 | 0.5658 |
| factor(X24)=0 | 4.5 | 363 | 0 | 1 | 0.5347 | 0.5052 | 0.5658 |
| factor(X24)=0 | 4.53 | 362 | 0 | 3 | 0.5347 | 0.5052 | 0.5658 |
| factor(X24)=0 | 4.54 | 359 | 0 | 1 | 0.5347 | 0.5052 | 0.5658 |
| factor(X24)=0 | 4.55 | 358 | 1 | 1 | 0.5332 | 0.5037 | 0.5644 |
| factor(X24)=0 | 4.58 | 356 | 0 | 2 | 0.5332 | 0.5037 | 0.5644 |
| factor(X24)=0 | 4.59 | 354 | 0 | 1 | 0.5332 | 0.5037 | 0.5644 |
| factor(X24)=0 | 4.6 | 353 | 0 | 4 | 0.5332 | 0.5037 | 0.5644 |
| factor(X24)=0 | 4.61 | 349 | 0 | 3 | 0.5332 | 0.5037 | 0.5644 |
| factor(X24)=0 | 4.62 | 346 | 0 | 1 | 0.5332 | 0.5037 | 0.5644 |
| factor(X24)=0 | 4.64 | 345 | 0 | 1 | 0.5332 | 0.5037 | 0.5644 |
| factor(X24)=0 | 4.65 | 344 | 0 | 1 | 0.5332 | 0.5037 | 0.5644 |
| factor(X24)=0 | 4.68 | 343 | 1 | 0 | 0.5316 | 0.5021 | 0.5629 |
| factor(X24)=0 | 4.7 | 342 | 0 | 1 | 0.5316 | 0.5021 | 0.5629 |
| factor(X24)=0 | 4.72 | 341 | 1 | 1 | 0.5300 | 0.5005 | 0.5614 |
| factor(X24)=0 | 4.73 | 339 | 0 | 2 | 0.5300 | 0.5005 | 0.5614 |
| factor(X24)=0 | 4.75 | 337 | 0 | 1 | 0.5300 | 0.5005 | 0.5614 |
| factor(X24)=0 | 4.79 | 336 | 0 | 1 | 0.5300 | 0.5005 | 0.5614 |
| factor(X24)=0 | 4.81 | 335 | 0 | 2 | 0.5300 | 0.5005 | 0.5614 |
| factor(X24)=0 | 4.82 | 333 | 0 | 1 | 0.5300 | 0.5005 | 0.5614 |
| factor(X24)=0 | 4.85 | 332 | 0 | 4 | 0.5300 | 0.5005 | 0.5614 |
| factor(X24)=0 | 4.88 | 328 | 0 | 1 | 0.5300 | 0.5005 | 0.5614 |
| factor(X24)=0 | 4.89 | 327 | 0 | 1 | 0.5300 | 0.5005 | 0.5614 |
| factor(X24)=0 | 4.9 | 326 | 0 | 1 | 0.5300 | 0.5005 | 0.5614 |
| factor(X24)=0 | 4.91 | 325 | 0 | 1 | 0.5300 | 0.5005 | 0.5614 |
| factor(X24)=0 | 4.94 | 324 | 0 | 2 | 0.5300 | 0.5005 | 0.5614 |
| factor(X24)=0 | 4.97 | 322 | 0 | 1 | 0.5300 | 0.5005 | 0.5614 |
| factor(X24)=0 | 4.99 | 321 | 0 | 1 | 0.5300 | 0.5005 | 0.5614 |
| factor(X24)=0 | 5 | 320 | 0 | 320 | 0.5300 | 0.5005 | 0.5614 |
| factor(X24)=1 | 0.13 | 132 | 2 | 0 | 0.9848 | 0.9642 | 1.0000 |
| factor(X24)=1 | 0.15 | 130 | 2 | 0 | 0.9697 | 0.9409 | 0.9994 |
| factor(X24)=1 | 0.16 | 128 | 5 | 0 | 0.9318 | 0.8898 | 0.9758 |
| factor(X24)=1 | 0.17 | 123 | 4 | 0 | 0.9015 | 0.8521 | 0.9538 |
| factor(X24)=1 | 0.18 | 119 | 2 | 0 | 0.8864 | 0.8338 | 0.9422 |
| factor(X24)=1 | 0.19 | 117 | 10 | 0 | 0.8106 | 0.7464 | 0.8803 |
| factor(X24)=1 | 0.2 | 107 | 3 | 0 | 0.7879 | 0.7211 | 0.8608 |
| factor(X24)=1 | 0.21 | 104 | 10 | 0 | 0.7121 | 0.6389 | 0.7937 |
| factor(X24)=1 | 0.22 | 94 | 9 | 0 | 0.6439 | 0.5672 | 0.7310 |
| factor(X24)=1 | 0.23 | 85 | 8 | 0 | 0.5833 | 0.5050 | 0.6738 |
| factor(X24)=1 | 0.24 | 77 | 3 | 0 | 0.5606 | 0.4820 | 0.6520 |
| factor(X24)=1 | 0.25 | 74 | 2 | 0 | 0.5455 | 0.4668 | 0.6374 |
| factor(X24)=1 | 0.27 | 72 | 1 | 0 | 0.5379 | 0.4592 | 0.6300 |
| factor(X24)=1 | 0.28 | 71 | 3 | 0 | 0.5152 | 0.4366 | 0.6079 |
| factor(X24)=1 | 0.29 | 68 | 1 | 0 | 0.5076 | 0.4291 | 0.6004 |
| factor(X24)=1 | 0.3 | 67 | 3 | 0 | 0.4848 | 0.4067 | 0.5781 |
| factor(X24)=1 | 0.32 | 64 | 1 | 0 | 0.4773 | 0.3992 | 0.5706 |
| factor(X24)=1 | 0.34 | 63 | 1 | 0 | 0.4697 | 0.3918 | 0.5630 |
| factor(X24)=1 | 0.47 | 62 | 2 | 0 | 0.4545 | 0.3771 | 0.5479 |
| factor(X24)=1 | 0.49 | 60 | 3 | 0 | 0.4318 | 0.3551 | 0.5252 |
| factor(X24)=1 | 0.5 | 57 | 1 | 0 | 0.4242 | 0.3478 | 0.5175 |
| factor(X24)=1 | 0.53 | 56 | 1 | 0 | 0.4167 | 0.3405 | 0.5099 |
| factor(X24)=1 | 0.59 | 55 | 1 | 0 | 0.4091 | 0.3333 | 0.5022 |
| factor(X24)=1 | 0.62 | 54 | 1 | 0 | 0.4015 | 0.3260 | 0.4945 |
| factor(X24)=1 | 0.64 | 53 | 3 | 0 | 0.3788 | 0.3045 | 0.4713 |
| factor(X24)=1 | 0.69 | 50 | 1 | 0 | 0.3712 | 0.2973 | 0.4635 |
| factor(X24)=1 | 0.72 | 49 | 1 | 0 | 0.3636 | 0.2902 | 0.4557 |
| factor(X24)=1 | 0.74 | 48 | 2 | 0 | 0.3485 | 0.2760 | 0.4400 |
| factor(X24)=1 | 0.77 | 46 | 2 | 0 | 0.3333 | 0.2619 | 0.4243 |
| factor(X24)=1 | 0.78 | 44 | 1 | 0 | 0.3258 | 0.2549 | 0.4164 |
| factor(X24)=1 | 0.86 | 43 | 0 | 1 | 0.3258 | 0.2549 | 0.4164 |
| factor(X24)=1 | 0.87 | 42 | 1 | 0 | 0.3180 | 0.2477 | 0.4083 |
| factor(X24)=1 | 0.91 | 41 | 1 | 0 | 0.3102 | 0.2405 | 0.4002 |
| factor(X24)=1 | 0.95 | 40 | 1 | 0 | 0.3025 | 0.2334 | 0.3921 |
| factor(X24)=1 | 1 | 39 | 1 | 0 | 0.2947 | 0.2263 | 0.3839 |
| factor(X24)=1 | 1.02 | 38 | 1 | 0 | 0.2870 | 0.2192 | 0.3757 |
| factor(X24)=1 | 1.07 | 37 | 1 | 0 | 0.2792 | 0.2121 | 0.3675 |
| factor(X24)=1 | 1.15 | 36 | 2 | 0 | 0.2637 | 0.1981 | 0.3510 |
| factor(X24)=1 | 1.17 | 34 | 1 | 0 | 0.2560 | 0.1912 | 0.3427 |
| factor(X24)=1 | 1.38 | 33 | 1 | 0 | 0.2482 | 0.1842 | 0.3344 |
| factor(X24)=1 | 1.42 | 32 | 3 | 0 | 0.2249 | 0.1636 | 0.3092 |
| factor(X24)=1 | 1.74 | 29 | 1 | 0 | 0.2172 | 0.1568 | 0.3007 |
| factor(X24)=1 | 2.26 | 28 | 1 | 0 | 0.2094 | 0.1501 | 0.2922 |
| factor(X24)=1 | 2.72 | 27 | 3 | 0 | 0.1861 | 0.1300 | 0.2665 |
| factor(X24)=1 | 3.39 | 24 | 2 | 0 | 0.1706 | 0.1168 | 0.2492 |
| factor(X24)=1 | 3.68 | 22 | 0 | 1 | 0.1706 | 0.1168 | 0.2492 |
| factor(X24)=1 | 4.61 | 21 | 0 | 1 | 0.1706 | 0.1168 | 0.2492 |
| factor(X24)=1 | 4.87 | 20 | 0 | 2 | 0.1706 | 0.1168 | 0.2492 |
| factor(X24)=1 | 5 | 18 | 0 | 18 | 0.1706 | 0.1168 | 0.2492 |

|  |  |  |  |  |  |  |  |  |  |
| --- | --- | --- | --- | --- | --- | --- | --- | --- | --- |
| X24 | records | n.max | n.start | events | \*rmean | \*se(rmean) | median | 0.95LCL | 0.95UCL |
| factor(X24)=0 | 1073 | 1073 | 1073 | 494 | 3.147 | 0.062 | NA | NA | NA |
| factor(X24)=1 | 132 | 132 | 132 | 109 | 1.319 | 0.155 | 0.3 | 0.24 | 0.62 |

Landmark analysis for time segment: EFS(years) > 5
Log rank test: implements the G-rho family of Harrington and Fleming (1982), with weights on each death of S(t)^rho, where S is the Kaplan-Meier estimate of survival. With rho = 0 this is the log-rank or Mantel-Haenszel test.

|  |  |  |  |
| --- | --- | --- | --- |
|  | N | Observed | Expected |
| factor(X24)=0 | 319 | 8 | 7.4194 |
| factor(X24)=1 | 18 | 0 | 0.5806 |

Chisq=
0.6304
on
1
degree of freedom, p=
0.4272
Survival table

|  |  |  |  |  |  |  |  |
| --- | --- | --- | --- | --- | --- | --- | --- |
|  | EFS(years) | N.Risk | N.Event | N.Censor | Survival | 95%CI Low | 95%CI Upp |
| factor(X24)=0 | 5.02 | 319 | 0 | 1 | 1.0000 | 1.0000 | 1.0000 |
| factor(X24)=0 | 5.03 | 318 | 0 | 1 | 1.0000 | 1.0000 | 1.0000 |
| factor(X24)=0 | 5.04 | 317 | 0 | 1 | 1.0000 | 1.0000 | 1.0000 |
| factor(X24)=0 | 5.05 | 316 | 0 | 2 | 1.0000 | 1.0000 | 1.0000 |
| factor(X24)=0 | 5.08 | 314 | 0 | 1 | 1.0000 | 1.0000 | 1.0000 |
| factor(X24)=0 | 5.1 | 313 | 0 | 1 | 1.0000 | 1.0000 | 1.0000 |
| factor(X24)=0 | 5.12 | 312 | 1 | 1 | 0.9968 | 0.9905 | 1.0000 |
| factor(X24)=0 | 5.14 | 310 | 0 | 1 | 0.9968 | 0.9905 | 1.0000 |
| factor(X24)=0 | 5.15 | 309 | 0 | 2 | 0.9968 | 0.9905 | 1.0000 |
| factor(X24)=0 | 5.16 | 307 | 0 | 1 | 0.9968 | 0.9905 | 1.0000 |
| factor(X24)=0 | 5.17 | 306 | 0 | 1 | 0.9968 | 0.9905 | 1.0000 |
| factor(X24)=0 | 5.18 | 305 | 0 | 3 | 0.9968 | 0.9905 | 1.0000 |
| factor(X24)=0 | 5.2 | 302 | 0 | 2 | 0.9968 | 0.9905 | 1.0000 |
| factor(X24)=0 | 5.22 | 300 | 0 | 1 | 0.9968 | 0.9905 | 1.0000 |
| factor(X24)=0 | 5.25 | 299 | 0 | 2 | 0.9968 | 0.9905 | 1.0000 |
| factor(X24)=0 | 5.26 | 297 | 0 | 2 | 0.9968 | 0.9905 | 1.0000 |
| factor(X24)=0 | 5.27 | 295 | 0 | 2 | 0.9968 | 0.9905 | 1.0000 |
| factor(X24)=0 | 5.29 | 293 | 0 | 2 | 0.9968 | 0.9905 | 1.0000 |
| factor(X24)=0 | 5.3 | 291 | 0 | 1 | 0.9968 | 0.9905 | 1.0000 |
| factor(X24)=0 | 5.31 | 290 | 0 | 1 | 0.9968 | 0.9905 | 1.0000 |
| factor(X24)=0 | 5.32 | 289 | 0 | 2 | 0.9968 | 0.9905 | 1.0000 |
| factor(X24)=0 | 5.33 | 287 | 0 | 2 | 0.9968 | 0.9905 | 1.0000 |
| factor(X24)=0 | 5.34 | 285 | 0 | 2 | 0.9968 | 0.9905 | 1.0000 |
| factor(X24)=0 | 5.35 | 283 | 0 | 6 | 0.9968 | 0.9905 | 1.0000 |
| factor(X24)=0 | 5.36 | 277 | 0 | 2 | 0.9968 | 0.9905 | 1.0000 |
| factor(X24)=0 | 5.37 | 275 | 0 | 2 | 0.9968 | 0.9905 | 1.0000 |
| factor(X24)=0 | 5.38 | 273 | 0 | 2 | 0.9968 | 0.9905 | 1.0000 |
| factor(X24)=0 | 5.39 | 271 | 0 | 1 | 0.9968 | 0.9905 | 1.0000 |
| factor(X24)=0 | 5.4 | 270 | 0 | 1 | 0.9968 | 0.9905 | 1.0000 |
| factor(X24)=0 | 5.41 | 269 | 0 | 2 | 0.9968 | 0.9905 | 1.0000 |
| factor(X24)=0 | 5.42 | 267 | 0 | 4 | 0.9968 | 0.9905 | 1.0000 |
| factor(X24)=0 | 5.43 | 263 | 0 | 1 | 0.9968 | 0.9905 | 1.0000 |
| factor(X24)=0 | 5.44 | 262 | 0 | 4 | 0.9968 | 0.9905 | 1.0000 |
| factor(X24)=0 | 5.45 | 258 | 0 | 1 | 0.9968 | 0.9905 | 1.0000 |
| factor(X24)=0 | 5.46 | 257 | 0 | 2 | 0.9968 | 0.9905 | 1.0000 |
| factor(X24)=0 | 5.48 | 255 | 0 | 3 | 0.9968 | 0.9905 | 1.0000 |
| factor(X24)=0 | 5.49 | 252 | 0 | 1 | 0.9968 | 0.9905 | 1.0000 |
| factor(X24)=0 | 5.5 | 251 | 0 | 1 | 0.9968 | 0.9905 | 1.0000 |
| factor(X24)=0 | 5.51 | 250 | 0 | 2 | 0.9968 | 0.9905 | 1.0000 |
| factor(X24)=0 | 5.52 | 248 | 0 | 2 | 0.9968 | 0.9905 | 1.0000 |
| factor(X24)=0 | 5.54 | 246 | 0 | 4 | 0.9968 | 0.9905 | 1.0000 |
| factor(X24)=0 | 5.55 | 242 | 0 | 2 | 0.9968 | 0.9905 | 1.0000 |
| factor(X24)=0 | 5.56 | 240 | 0 | 2 | 0.9968 | 0.9905 | 1.0000 |
| factor(X24)=0 | 5.57 | 238 | 0 | 1 | 0.9968 | 0.9905 | 1.0000 |
| factor(X24)=0 | 5.58 | 237 | 1 | 1 | 0.9926 | 0.9823 | 1.0000 |
| factor(X24)=0 | 5.6 | 235 | 0 | 1 | 0.9926 | 0.9823 | 1.0000 |
| factor(X24)=0 | 5.61 | 234 | 0 | 1 | 0.9926 | 0.9823 | 1.0000 |
| factor(X24)=0 | 5.63 | 233 | 0 | 2 | 0.9926 | 0.9823 | 1.0000 |
| factor(X24)=0 | 5.65 | 231 | 0 | 1 | 0.9926 | 0.9823 | 1.0000 |
| factor(X24)=0 | 5.66 | 230 | 0 | 1 | 0.9926 | 0.9823 | 1.0000 |
| factor(X24)=0 | 5.67 | 229 | 0 | 3 | 0.9926 | 0.9823 | 1.0000 |
| factor(X24)=0 | 5.7 | 226 | 0 | 3 | 0.9926 | 0.9823 | 1.0000 |
| factor(X24)=0 | 5.71 | 223 | 0 | 2 | 0.9926 | 0.9823 | 1.0000 |
| factor(X24)=0 | 5.72 | 221 | 0 | 1 | 0.9926 | 0.9823 | 1.0000 |
| factor(X24)=0 | 5.73 | 220 | 0 | 2 | 0.9926 | 0.9823 | 1.0000 |
| factor(X24)=0 | 5.74 | 218 | 0 | 1 | 0.9926 | 0.9823 | 1.0000 |
| factor(X24)=0 | 5.75 | 217 | 0 | 2 | 0.9926 | 0.9823 | 1.0000 |
| factor(X24)=0 | 5.77 | 215 | 0 | 1 | 0.9926 | 0.9823 | 1.0000 |
| factor(X24)=0 | 5.78 | 214 | 0 | 2 | 0.9926 | 0.9823 | 1.0000 |
| factor(X24)=0 | 5.79 | 212 | 0 | 1 | 0.9926 | 0.9823 | 1.0000 |
| factor(X24)=0 | 5.8 | 211 | 0 | 1 | 0.9926 | 0.9823 | 1.0000 |
| factor(X24)=0 | 5.81 | 210 | 1 | 2 | 0.9879 | 0.9741 | 1.0000 |
| factor(X24)=0 | 5.84 | 207 | 0 | 2 | 0.9879 | 0.9741 | 1.0000 |
| factor(X24)=0 | 5.85 | 205 | 0 | 1 | 0.9879 | 0.9741 | 1.0000 |
| factor(X24)=0 | 5.88 | 204 | 0 | 1 | 0.9879 | 0.9741 | 1.0000 |
| factor(X24)=0 | 5.89 | 203 | 0 | 2 | 0.9879 | 0.9741 | 1.0000 |
| factor(X24)=0 | 5.92 | 201 | 0 | 1 | 0.9879 | 0.9741 | 1.0000 |
| factor(X24)=0 | 5.93 | 200 | 0 | 1 | 0.9879 | 0.9741 | 1.0000 |
| factor(X24)=0 | 5.94 | 199 | 0 | 2 | 0.9879 | 0.9741 | 1.0000 |
| factor(X24)=0 | 5.95 | 197 | 0 | 3 | 0.9879 | 0.9741 | 1.0000 |
| factor(X24)=0 | 5.96 | 194 | 0 | 1 | 0.9879 | 0.9741 | 1.0000 |
| factor(X24)=0 | 5.98 | 193 | 0 | 1 | 0.9879 | 0.9741 | 1.0000 |
| factor(X24)=0 | 6 | 192 | 0 | 6 | 0.9879 | 0.9741 | 1.0000 |
| factor(X24)=0 | 6.01 | 186 | 0 | 1 | 0.9879 | 0.9741 | 1.0000 |
| factor(X24)=0 | 6.02 | 185 | 0 | 1 | 0.9879 | 0.9741 | 1.0000 |
| factor(X24)=0 | 6.04 | 184 | 0 | 2 | 0.9879 | 0.9741 | 1.0000 |
| factor(X24)=0 | 6.07 | 182 | 0 | 1 | 0.9879 | 0.9741 | 1.0000 |
| factor(X24)=0 | 6.08 | 181 | 0 | 1 | 0.9879 | 0.9741 | 1.0000 |
| factor(X24)=0 | 6.12 | 180 | 0 | 2 | 0.9879 | 0.9741 | 1.0000 |
| factor(X24)=0 | 6.15 | 178 | 0 | 3 | 0.9879 | 0.9741 | 1.0000 |
| factor(X24)=0 | 6.16 | 175 | 0 | 2 | 0.9879 | 0.9741 | 1.0000 |
| factor(X24)=0 | 6.17 | 173 | 0 | 1 | 0.9879 | 0.9741 | 1.0000 |
| factor(X24)=0 | 6.18 | 172 | 0 | 2 | 0.9879 | 0.9741 | 1.0000 |
| factor(X24)=0 | 6.22 | 170 | 0 | 1 | 0.9879 | 0.9741 | 1.0000 |
| factor(X24)=0 | 6.25 | 169 | 0 | 2 | 0.9879 | 0.9741 | 1.0000 |
| factor(X24)=0 | 6.27 | 167 | 0 | 1 | 0.9879 | 0.9741 | 1.0000 |
| factor(X24)=0 | 6.28 | 166 | 0 | 1 | 0.9879 | 0.9741 | 1.0000 |
| factor(X24)=0 | 6.29 | 165 | 0 | 1 | 0.9879 | 0.9741 | 1.0000 |
| factor(X24)=0 | 6.32 | 164 | 0 | 1 | 0.9879 | 0.9741 | 1.0000 |
| factor(X24)=0 | 6.35 | 163 | 0 | 2 | 0.9879 | 0.9741 | 1.0000 |
| factor(X24)=0 | 6.36 | 161 | 0 | 1 | 0.9879 | 0.9741 | 1.0000 |
| factor(X24)=0 | 6.37 | 160 | 0 | 2 | 0.9879 | 0.9741 | 1.0000 |
| factor(X24)=0 | 6.38 | 158 | 0 | 2 | 0.9879 | 0.9741 | 1.0000 |
| factor(X24)=0 | 6.39 | 156 | 0 | 4 | 0.9879 | 0.9741 | 1.0000 |
| factor(X24)=0 | 6.4 | 152 | 0 | 2 | 0.9879 | 0.9741 | 1.0000 |
| factor(X24)=0 | 6.42 | 150 | 0 | 3 | 0.9879 | 0.9741 | 1.0000 |
| factor(X24)=0 | 6.45 | 147 | 3 | 1 | 0.9677 | 0.9417 | 0.9944 |
| factor(X24)=0 | 6.46 | 143 | 0 | 2 | 0.9677 | 0.9417 | 0.9944 |
| factor(X24)=0 | 6.47 | 141 | 0 | 3 | 0.9677 | 0.9417 | 0.9944 |
| factor(X24)=0 | 6.48 | 138 | 0 | 2 | 0.9677 | 0.9417 | 0.9944 |
| factor(X24)=0 | 6.49 | 136 | 0 | 1 | 0.9677 | 0.9417 | 0.9944 |
| factor(X24)=0 | 6.5 | 135 | 0 | 2 | 0.9677 | 0.9417 | 0.9944 |
| factor(X24)=0 | 6.51 | 133 | 0 | 2 | 0.9677 | 0.9417 | 0.9944 |
| factor(X24)=0 | 6.52 | 131 | 0 | 1 | 0.9677 | 0.9417 | 0.9944 |
| factor(X24)=0 | 6.53 | 130 | 0 | 1 | 0.9677 | 0.9417 | 0.9944 |
| factor(X24)=0 | 6.54 | 129 | 0 | 1 | 0.9677 | 0.9417 | 0.9944 |
| factor(X24)=0 | 6.55 | 128 | 0 | 1 | 0.9677 | 0.9417 | 0.9944 |
| factor(X24)=0 | 6.56 | 127 | 1 | 1 | 0.9601 | 0.9305 | 0.9906 |
| factor(X24)=0 | 6.57 | 125 | 0 | 2 | 0.9601 | 0.9305 | 0.9906 |
| factor(X24)=0 | 6.58 | 123 | 0 | 2 | 0.9601 | 0.9305 | 0.9906 |
| factor(X24)=0 | 6.59 | 121 | 0 | 1 | 0.9601 | 0.9305 | 0.9906 |
| factor(X24)=0 | 6.6 | 120 | 0 | 1 | 0.9601 | 0.9305 | 0.9906 |
| factor(X24)=0 | 6.63 | 119 | 0 | 1 | 0.9601 | 0.9305 | 0.9906 |
| factor(X24)=0 | 6.64 | 118 | 0 | 2 | 0.9601 | 0.9305 | 0.9906 |
| factor(X24)=0 | 6.65 | 116 | 0 | 3 | 0.9601 | 0.9305 | 0.9906 |
| factor(X24)=0 | 6.68 | 113 | 0 | 1 | 0.9601 | 0.9305 | 0.9906 |
| factor(X24)=0 | 6.69 | 112 | 0 | 1 | 0.9601 | 0.9305 | 0.9906 |
| factor(X24)=0 | 6.73 | 111 | 0 | 1 | 0.9601 | 0.9305 | 0.9906 |
| factor(X24)=0 | 6.75 | 110 | 0 | 3 | 0.9601 | 0.9305 | 0.9906 |
| factor(X24)=0 | 6.77 | 107 | 0 | 1 | 0.9601 | 0.9305 | 0.9906 |
| factor(X24)=0 | 6.78 | 106 | 0 | 1 | 0.9601 | 0.9305 | 0.9906 |
| factor(X24)=0 | 6.79 | 105 | 0 | 2 | 0.9601 | 0.9305 | 0.9906 |
| factor(X24)=0 | 6.81 | 103 | 0 | 2 | 0.9601 | 0.9305 | 0.9906 |
| factor(X24)=0 | 6.85 | 101 | 0 | 1 | 0.9601 | 0.9305 | 0.9906 |
| factor(X24)=0 | 6.87 | 100 | 0 | 1 | 0.9601 | 0.9305 | 0.9906 |
| factor(X24)=0 | 6.89 | 99 | 0 | 2 | 0.9601 | 0.9305 | 0.9906 |
| factor(X24)=0 | 6.91 | 97 | 0 | 1 | 0.9601 | 0.9305 | 0.9906 |
| factor(X24)=0 | 6.92 | 96 | 0 | 1 | 0.9601 | 0.9305 | 0.9906 |
| factor(X24)=0 | 6.94 | 95 | 0 | 1 | 0.9601 | 0.9305 | 0.9906 |
| factor(X24)=0 | 6.96 | 94 | 0 | 4 | 0.9601 | 0.9305 | 0.9906 |
| factor(X24)=0 | 6.98 | 90 | 0 | 1 | 0.9601 | 0.9305 | 0.9906 |
| factor(X24)=0 | 7 | 89 | 0 | 1 | 0.9601 | 0.9305 | 0.9906 |
| factor(X24)=0 | 7.08 | 88 | 0 | 1 | 0.9601 | 0.9305 | 0.9906 |
| factor(X24)=0 | 7.14 | 87 | 0 | 1 | 0.9601 | 0.9305 | 0.9906 |
| factor(X24)=0 | 7.16 | 86 | 0 | 1 | 0.9601 | 0.9305 | 0.9906 |
| factor(X24)=0 | 7.2 | 85 | 0 | 1 | 0.9601 | 0.9305 | 0.9906 |
| factor(X24)=0 | 7.21 | 84 | 0 | 1 | 0.9601 | 0.9305 | 0.9906 |
| factor(X24)=0 | 7.26 | 83 | 0 | 3 | 0.9601 | 0.9305 | 0.9906 |
| factor(X24)=0 | 7.29 | 80 | 0 | 1 | 0.9601 | 0.9305 | 0.9906 |
| factor(X24)=0 | 7.35 | 79 | 0 | 2 | 0.9601 | 0.9305 | 0.9906 |
| factor(X24)=0 | 7.36 | 77 | 0 | 6 | 0.9601 | 0.9305 | 0.9906 |
| factor(X24)=0 | 7.37 | 71 | 0 | 1 | 0.9601 | 0.9305 | 0.9906 |
| factor(X24)=0 | 7.38 | 70 | 0 | 1 | 0.9601 | 0.9305 | 0.9906 |
| factor(X24)=0 | 7.39 | 69 | 0 | 2 | 0.9601 | 0.9305 | 0.9906 |
| factor(X24)=0 | 7.4 | 67 | 0 | 1 | 0.9601 | 0.9305 | 0.9906 |
| factor(X24)=0 | 7.41 | 66 | 0 | 1 | 0.9601 | 0.9305 | 0.9906 |
| factor(X24)=0 | 7.42 | 65 | 0 | 1 | 0.9601 | 0.9305 | 0.9906 |
| factor(X24)=0 | 7.45 | 64 | 0 | 1 | 0.9601 | 0.9305 | 0.9906 |
| factor(X24)=0 | 7.47 | 63 | 0 | 1 | 0.9601 | 0.9305 | 0.9906 |
| factor(X24)=0 | 7.48 | 62 | 0 | 3 | 0.9601 | 0.9305 | 0.9906 |
| factor(X24)=0 | 7.5 | 59 | 0 | 1 | 0.9601 | 0.9305 | 0.9906 |
| factor(X24)=0 | 7.53 | 58 | 0 | 1 | 0.9601 | 0.9305 | 0.9906 |
| factor(X24)=0 | 7.54 | 57 | 0 | 1 | 0.9601 | 0.9305 | 0.9906 |
| factor(X24)=0 | 7.55 | 56 | 0 | 1 | 0.9601 | 0.9305 | 0.9906 |
| factor(X24)=0 | 7.56 | 55 | 0 | 2 | 0.9601 | 0.9305 | 0.9906 |
| factor(X24)=0 | 7.58 | 53 | 0 | 4 | 0.9601 | 0.9305 | 0.9906 |
| factor(X24)=0 | 7.59 | 49 | 0 | 1 | 0.9601 | 0.9305 | 0.9906 |
| factor(X24)=0 | 7.6 | 48 | 0 | 2 | 0.9601 | 0.9305 | 0.9906 |
| factor(X24)=0 | 7.61 | 46 | 0 | 1 | 0.9601 | 0.9305 | 0.9906 |
| factor(X24)=0 | 7.62 | 45 | 0 | 1 | 0.9601 | 0.9305 | 0.9906 |
| factor(X24)=0 | 7.67 | 44 | 0 | 1 | 0.9601 | 0.9305 | 0.9906 |
| factor(X24)=0 | 7.68 | 43 | 0 | 1 | 0.9601 | 0.9305 | 0.9906 |
| factor(X24)=0 | 7.69 | 42 | 0 | 2 | 0.9601 | 0.9305 | 0.9906 |
| factor(X24)=0 | 7.73 | 40 | 0 | 2 | 0.9601 | 0.9305 | 0.9906 |
| factor(X24)=0 | 7.74 | 38 | 0 | 3 | 0.9601 | 0.9305 | 0.9906 |
| factor(X24)=0 | 7.81 | 35 | 0 | 1 | 0.9601 | 0.9305 | 0.9906 |
| factor(X24)=0 | 7.82 | 34 | 0 | 1 | 0.9601 | 0.9305 | 0.9906 |
| factor(X24)=0 | 7.9 | 33 | 0 | 1 | 0.9601 | 0.9305 | 0.9906 |
| factor(X24)=0 | 7.94 | 32 | 0 | 2 | 0.9601 | 0.9305 | 0.9906 |
| factor(X24)=0 | 7.98 | 30 | 0 | 1 | 0.9601 | 0.9305 | 0.9906 |
| factor(X24)=0 | 7.99 | 29 | 1 | 1 | 0.9270 | 0.8595 | 0.9997 |
| factor(X24)=0 | 8.01 | 27 | 0 | 1 | 0.9270 | 0.8595 | 0.9997 |
| factor(X24)=0 | 8.06 | 26 | 0 | 1 | 0.9270 | 0.8595 | 0.9997 |
| factor(X24)=0 | 8.07 | 25 | 0 | 1 | 0.9270 | 0.8595 | 0.9997 |
| factor(X24)=0 | 8.17 | 24 | 0 | 1 | 0.9270 | 0.8595 | 0.9997 |
| factor(X24)=0 | 8.21 | 23 | 0 | 1 | 0.9270 | 0.8595 | 0.9997 |
| factor(X24)=0 | 8.26 | 22 | 0 | 1 | 0.9270 | 0.8595 | 0.9997 |
| factor(X24)=0 | 8.27 | 21 | 0 | 1 | 0.9270 | 0.8595 | 0.9997 |
| factor(X24)=0 | 8.28 | 20 | 0 | 1 | 0.9270 | 0.8595 | 0.9997 |
| factor(X24)=0 | 8.29 | 19 | 0 | 1 | 0.9270 | 0.8595 | 0.9997 |
| factor(X24)=0 | 8.36 | 18 | 0 | 1 | 0.9270 | 0.8595 | 0.9997 |
| factor(X24)=0 | 8.48 | 17 | 0 | 1 | 0.9270 | 0.8595 | 0.9997 |
| factor(X24)=0 | 8.49 | 16 | 0 | 2 | 0.9270 | 0.8595 | 0.9997 |
| factor(X24)=0 | 8.5 | 14 | 0 | 1 | 0.9270 | 0.8595 | 0.9997 |
| factor(X24)=0 | 8.53 | 13 | 0 | 1 | 0.9270 | 0.8595 | 0.9997 |
| factor(X24)=0 | 8.72 | 12 | 0 | 1 | 0.9270 | 0.8595 | 0.9997 |
| factor(X24)=0 | 8.84 | 11 | 0 | 1 | 0.9270 | 0.8595 | 0.9997 |
| factor(X24)=0 | 8.88 | 10 | 0 | 3 | 0.9270 | 0.8595 | 0.9997 |
| factor(X24)=0 | 9.04 | 7 | 0 | 1 | 0.9270 | 0.8595 | 0.9997 |
| factor(X24)=0 | 9.06 | 6 | 0 | 2 | 0.9270 | 0.8595 | 0.9997 |
| factor(X24)=0 | 9.08 | 4 | 0 | 1 | 0.9270 | 0.8595 | 0.9997 |
| factor(X24)=0 | 9.26 | 3 | 0 | 1 | 0.9270 | 0.8595 | 0.9997 |
| factor(X24)=0 | 9.61 | 2 | 0 | 1 | 0.9270 | 0.8595 | 0.9997 |
| factor(X24)=0 | 9.95 | 1 | 0 | 1 | 0.9270 | 0.8595 | 0.9997 |
| factor(X24)=1 | 5.33 | 18 | 0 | 1 | 1.0000 | 1.0000 | 1.0000 |
| factor(X24)=1 | 5.38 | 17 | 0 | 1 | 1.0000 | 1.0000 | 1.0000 |
| factor(X24)=1 | 5.4 | 16 | 0 | 1 | 1.0000 | 1.0000 | 1.0000 |
| factor(X24)=1 | 6.19 | 15 | 0 | 1 | 1.0000 | 1.0000 | 1.0000 |
| factor(X24)=1 | 6.2 | 14 | 0 | 1 | 1.0000 | 1.0000 | 1.0000 |
| factor(X24)=1 | 6.4 | 13 | 0 | 1 | 1.0000 | 1.0000 | 1.0000 |
| factor(X24)=1 | 6.51 | 12 | 0 | 1 | 1.0000 | 1.0000 | 1.0000 |
| factor(X24)=1 | 7 | 11 | 0 | 2 | 1.0000 | 1.0000 | 1.0000 |
| factor(X24)=1 | 7.12 | 9 | 0 | 1 | 1.0000 | 1.0000 | 1.0000 |
| factor(X24)=1 | 7.27 | 8 | 0 | 1 | 1.0000 | 1.0000 | 1.0000 |
| factor(X24)=1 | 7.46 | 7 | 0 | 1 | 1.0000 | 1.0000 | 1.0000 |
| factor(X24)=1 | 7.72 | 6 | 0 | 1 | 1.0000 | 1.0000 | 1.0000 |
| factor(X24)=1 | 7.74 | 5 | 0 | 2 | 1.0000 | 1.0000 | 1.0000 |
| factor(X24)=1 | 8.1 | 3 | 0 | 1 | 1.0000 | 1.0000 | 1.0000 |
| factor(X24)=1 | 8.15 | 2 | 0 | 1 | 1.0000 | 1.0000 | 1.0000 |
| factor(X24)=1 | 8.69 | 1 | 0 | 1 | 1.0000 | 1.0000 | 1.0000 |

|  |  |  |  |  |  |  |  |  |  |
| --- | --- | --- | --- | --- | --- | --- | --- | --- | --- |
| X24 | records | n.max | n.start | events | \*rmean | \*se(rmean) | median | 0.95LCL | 0.95UCL |
| factor(X24)=0 | 319 | 319 | 319 | 8 | 9.151 | 0.063 | NA | NA | NA |
| factor(X24)=1 | 18 | 18 | 18 | 0 | 9.32 | 0 | NA | NA | NA |

Created by EmpowerStats (www.empowerstats.com) and R on 2025-10-07
